# Changes in triggering of ST-elevation myocardial infarction by particulate air pollution in Monroe County, New York over time: a case-crossover study

**DOI:** 10.1186/s12940-019-0521-3

**Published:** 2019-09-06

**Authors:** Meng Wang, Philip K. Hopke, Mauro Masiol, Sally W. Thurston, Scott Cameron, Frederick Ling, Edwin van Wijngaarden, Daniel Croft, Stefania Squizzato, Kelly Thevenet-Morrison, David Chalupa, David Q. Rich

**Affiliations:** 1 Department of Public Health Sciences, University of Rochester Medical Center, Rochester, NY USA; 2 Center for Air Resources Engineering and Science, Clarkson University, Potsdam, NY USA; 3 Department of Biostatistics and Computational Biology, University of Rochester Medical Center, Rochester, NY USA; 4 Department of Medicine, University of Rochester Medical Center, Rochester, NY USA; 5 Department of Environmental Medicine, University of Rochester Medical Center, Rochester, NY USA; 6 Department of Public Health Sciences, University of Rochester School of Medicine and Dentistry, 265 Crittenden Boulevard, CU 420644, Rochester, NY 14642 USA

**Keywords:** Particulate matter, Ultrafine particles, ST elevation myocardial infarction, Air quality, Effect modification, Case-crossover

## Abstract

**Background:**

Previous studies have reported that fine particle (PM_2.5_) concentrations triggered ST elevation myocardial infarctions (STEMI). In Rochester, NY, multiple air quality policies and economic changes/influences from 2008 to 2013 led to decreased concentrations of PM_2.5_ and its major constituents (SO_4_^2−^, NO_3_^−^, elemental and primary organic carbon). This study examined whether the rate of STEMI associated with increased ambient gaseous and PM component concentrations was different AFTER these air quality policies and economic changes (2014–2016), compared to DURING (2008–2013) and BEFORE these polices and changes (2005–2007).

**Methods:**

Using 921 STEMIs treated at the University of Rochester Medical Center (2005–2016) and a case-crossover design, we examined whether the rate of STEMI associated with increased PM_2.5_, ultrafine particles (UFP, < 100 nm), accumulation mode particles (AMP, 100-500 nm), black carbon, SO_2_, CO, and O_3_ concentrations in the previous 1–72 h was modified by the time period related to these pollutant source changes (BEFORE, DURING, AFTER).

**Results:**

Each interquartile range (3702 particles/cm^3^) increase in UFP concentration in the previous 1 h was associated with a 12% (95% CI = 3%, 22%) increase in the rate of STEMI. The effect size was larger in the AFTER period (26%) than the DURING (5%) or BEFORE periods (9%). There were similar patterns for black carbon and SO_2_.

**Conclusions:**

An increased rate of STEMI associated with UFP and other pollutant concentrations was higher in the AFTER period compared to the BEFORE and DURING periods. This may be due to changes in PM composition (e.g. higher secondary organic carbon and particle bound reactive oxygen species) following these air quality policies and economic changes.

**Electronic supplementary material:**

The online version of this article (10.1186/s12940-019-0521-3) contains supplementary material, which is available to authorized users.

## Introduction

Over the past decade, policy initiatives to improve air quality have been implemented nationwide and in New York State [1]. These measures included the lowering of the sulfur content of diesel and home heating fuels, use of particle regenerative traps, and nitrogen oxide controls on new heavy-duty diesel vehicles. Additionally, several actions to reduce sulfur dioxide (SO_2)_ and nitrogen oxide (NOx) emissions from power plants in upwind source areas have also been enacted (i.e. Cross-State Air Pollution Rule). Furthermore, economic changes have also led to changes in emissions [1, 2]. The economic drivers include the recession of 2008 that resulted in generally decreased economic activity and thus lower emissions, and a dramatic decline in the price of natural gas that displaced coal as a fuel for electricity generation.

These policy initiatives and economic drivers resulted in changes in the concentrations and composition of air pollutants measured in Rochester, NY [1–3]. Long-term trend analyses from 2001/2002 to 2015/2016 in Rochester showed decreases in concentrations of particulate matter with aerodynamic diameter < 2.5 μm (PM_2.5_), accumulation mode particles (AMP, 100–500 nm), ultrafine particles (UFP, ≤ 100 nm), black carbon (BC), SO_2_, and carbon monoxide (CO), while the concentration of ozone (O_3_) showed a slight upward trend [4]. The reduction in PM_2.5_ concentrations was largely attributed to substantial decreases in sulfate and nitrate (− 65% and − 37%, respectively, from 2001 to 2015) [1], which are secondary PM species formed from the oxidation of precursor gases SO_2_ and NOx. These pollutants have been previously associated with adverse cardiovascular outcomes [5–10].

Short-term increases in PM_2.5_ concentrations in the previous hours and days have been repeatedly associated with the triggering of myocardial infarction [8, 11–17], including prior Rochester studies reporting triggering of ST segment elevation myocardial infarction (STEMI) by short-term increases in ambient PM_2.5_ concentrations in the previous hour [11, 12]. Furthermore, Rich et al. reported a higher rate of MI associated with increased PM_2.5_ concentrations when the PM_2.5_ mixtures had high mass fractions of secondary PM species (sulfate, nitrate, and/or organics), compared to when the mixtures had low mass fractions of secondary PM species [18]. This result suggests that PM_2.5_ high in secondary species, formed through active, oxidative atmospheric photochemistry, may be more likely to trigger myocardial infarctions than primary fine particles [18]. Given these findings, reduced concentrations of secondary PM species sulfate and nitrate following the policy initiatives described above may result in a lower rate of STEMI per unit mass of PM, compared to periods when PM contained a higher proportion of secondary PM.

Using data on patients with a STEMI treated at the University of Rochester Medical Center (URMC) and air pollutant concentrations measured in Rochester, we examined whether the rate of STEMI associated with increased PM (PM_2.5_, UFP, and AMP) concentrations in the previous few hours and days was modified by periods defined based on the time of pollutant source changes following these environmental policies. We hypothesized that increases in the rate of STEMI associated with the same incremental increase in PM concentrations would be smaller AFTER these pollutant changes occurred (2014–2016), compared to BEFORE (2005–2007), and DURING this period (2008–2013). We also explored effect modifications by periods (BEFORE, DURING, and AFTER) of the rate of STEMI associated with concentrations of BC and gaseous pollutants SO_2_, O_3_, and CO.

## Methods

### Study population

We included all patients treated at the Cardiac Catheterization Laboratory (Cath Lab) at URMC in Rochester, NY for STEMI between January 1, 2005 and December 31, 2016, who resided within 15 miles of the pollution monitoring station in Rochester. If a patient experienced multiple STEMIs during the study period, we only included STEMI that occurred at least 72 h after a previous STEMI, resulting in 921 STEMI events in 912 patients available for analyses. Patients’ demographic and clinical characteristics were also obtained. This study was approved by the University of Rochester Medical Center Research Subjects Review Board. Informed consent was not required since data were past events and the research could not affect treatment, there was no contact with study subjects, and all results are presented in aggregate only.

### Outcome assessments

American College of Cardiology (ACC)/American Heart Association (AHA) guidelines were used at the time of Cath Lab admission to diagnose STEMI [19]. Specifically, STEMI was defined as ST segment elevation on the presenting electrocardiogram (ECG) of > 1 mm in ≥2 contiguous precordial leads, or in ≥2 adjacent limb leads, or new or presumed new left bundle branch block in the presence of angina or angina equivalent. ECG criteria are both necessary and sufficient for the diagnosis of STEMI in a patient deemed by the treating physician to have symptoms consistent with cardiac chest pain. Symptom onset time (date and hour) was self-reported by each patient (or kin if the patient was unable to communicate) upon arrival to the URMC Cath Lab.

### Air pollution and meteorology measurements

The New York State Department of Environmental Conservation operates a routine air quality monitoring site in Rochester, New York, where PM _2.5_ mass concentrations, SO_2_, O_3_, and CO were measured continuously throughout the study period (2005–2016). However, the CO and SO_2_ monitors were replaced near the end of 2010 with higher sensitivity models. The measured CO concentrations from 2007 to the installation of the new monitor were generally close to the instrument’s detection limit (0.2 ppm). This site operated a single-wavelength (880 nm) aethalometer (Magee Scientific, Berkeley, CA, USA) to measure black carbon (BC) until mid-2008, and a two-wavelength unit (370 and 880 nm) from July 2008 to the present. Particle number size distributions were measured using a Scanning Mobility Particle Sizer (SMPS, TSI, Inc., Shoreview, MN). The concentrations were aggregated into several size groups including UFP (≤ 100 nm) and AMP (100–500 nm). The UFP were further divided into UFP11-50 nm (marker for nucleation and spark ignition vehicle emissions) [20] and UFP50-100 nm (marker of diesel vehicle emissions and residential wood burning; Aitken mode) [21–23]. These data have been presented and their trends analyzed in several recent studies [3, 24]. NOx data were not available for the entire study period and thus were not included in our study. Temperature and relative humidity data measured at the Rochester International Airport were obtained from the National Climate Data Center. Hourly means of each pollutant and weather variables were used in all statistical analyses described below.

### Study design

We used a time-stratified case-crossover design to estimate the rate of STEMI associated with air pollutant concentrations [25, 26]. This design contrasts pollutant concentrations immediately before the acute STEMI (case-period) to other time periods when the patient did not have an acute STEMI (control period). Case periods of this study were defined as the 1, 3, 12, 24, 48, and 72-h periods prior to the time of STEMI symptom onset, with control periods (3–4 per case, depending on the number of days in calendar month) matched by calendar year, month, weekday, and hour of the day. Since these case and control periods were separated by 1 week, there was no overlap in the air pollutant concentrations included in the case and control periods. Further, since the case and control periods were from the same patient, time-invariant confounders such as age, gender, and chronic comorbidities were controlled by design. Factors that varied between the case and control periods (e.g., temperature and relative humidity) were potential confounders included in our models.

### Statistical analysis

First, we computed the average concentrations of each pollutant (PM_2.5_, AMP, UFP, UFP 11-50 nm, UFP 50-100 nm, BC, SO_2_, O_3_, and CO) in the 1 (lag hour 0), 3 (lag hours 0–2), 12 (lag hours 0–11), 24 (lag hours 0–23), 48 (lag hours 0–47), and 72 (lag hours 0–71) hours prior to each symptom onset (i.e. case) time and its matched control time. If the time of symptom onset of a STEMI event was estimated to be in the first 29 min of the hour (e.g. 12:28), then lag hour 0 was defined as the previous hour (i.e. 11:00–11:59). If the STEMI symptom onset time was estimated to be in the 30th minute or after (e.g. 12:49), then lag hour 0 was defined as that same hour (i.e. 12:00–12:59). We then regressed the case-control status (i.e., case = 1, control = 0) against the mean PM_2.5_ concentration at lag hour 0 of the case and control periods, using conditional logistic regression models stratified by each case-control set, adjusting for mean temperature and relative humidity during the same lag hours. We used Akaike’s information criterion to select the optimal functional form (natural spline with 2, 3, 4, or 5 degrees of freedom versus 1 degree of freedom/linear) for these two covariates. One degree of freedom (linear) was selected for both variables and used in the model. We also estimated the rate of STEMI associated with each interquartile (IQR) increase in the average PM_2.5_ concentration at longer lag hours (lag hours 0–2, 0–11, 0–23, 0–47, and 0–71) in separate models in the same manner. We then repeated these analyses for the other pollutants (AMP, UFP, UFP 11-50 nm, UFP 50-100 nm, BC, SO_2_, O_3_, CO). Three sensitivity analyses were performed. First, we examined whether the exposure-response functions (i.e. relative rates in the model described above) were linear or non-linear, by comparing AIC from the model described above, to models where the rate of STEMI associated with increased pollutant concentration was modeled using a natural spline with 2, 3, 4, or 5 degrees of freedom. Second, we examined whether the pollutant/STEMI association changed after further adjustment for holiday in the model. Third, we examined whether the pollutant/STEMI association was modified by age (< 65 vs. ≥65 years), sex (male vs. female), and history of smoking, diabetes, dyslipidemia, and heart failure (yes vs. no) in separate models by adding an interaction term (e.g. UFP *male) to the model described above. The IQRs used to scale effect estimates were the IQRs of the pollutant specific lag times, from the control periods during the entire study years (2005–2016). Since the correlation among data resulting from multiple STEMI events contributed by the same patient is likely negligible (921 events contributed by 912 patients), we did not account for within-subject correlation in our analyses.

To examine whether the rate of STEMI associated with increased PM_2.5_ concentration in the previous hour (lag hour 0) was modified by the period of pollutant source changes (BEFORE [2005–2007], DURING [2008–2013], and AFTER [2014–2016]), we added two interaction terms of ‘period’ (a categorical variable with three levels) and PM_2.5_ concentration to the model of main effect analysis described above. We then repeated this analysis for other lag hours (lag hours 0–2, 0–11, 0–23, 0–47, and 0–71) and the other pollutants (AMP, UFP, UFP 11-50 nm, UFP 50-100 nm, BC, SO_2_, O_3_, CO). To account for the unbalanced distribution of patients’ age across the three periods, in sensitivity analysis, we added an interaction term between pollutant concentration and age (< 65 years vs. ≥65 years) to the model of effect modification by period described above. We then repeated this analysis for all patient characteristics with unbalanced distributions across the three periods (sex, smoking, diabetes, dyslipidemia, heart failure). Analyses were performed using SAS (version 9.4; SAS institute Inc., Cary, NC) and R (version 3.0.1, R foundation for statistical computing, Vienna, Austria, *splines* package). A *p* < 0.05 was used to define statistical significance.

## Results

Most STEMI patients were male (71%) and non-Hispanic white (83%), and were on average 63 years of age (standard deviation: ± 14 years) (Table 1). Thirty-one percent of patients were smokers. Patients in the BEFORE period were generally older, less often male, and less often smokers compared to patients in the DURING and AFTER periods. The prevalence of a prior history of coronary artery bypass grafting (CABG), diabetes, and heart failure was higher in patients in the BEFORE period, than other periods. However, the prevalence of dyslipidemia was lower in patients in the BEFORE period (Table 1).

**Table 1:** Characteristics of study subjects, by period

Characteristic	All Years 2005–2016 (*N* = 921)^a^ n (%)	Before 2005–2007 (*N* = 249)^a^ n (%)	During 2008–2013 (*N* = 372)^a^ n (%)	After 2014–2016 (*N* = 300)^a^ n (%)
Age, years				
Missing ^b^	4 (0)	2(1)	2(1)	0(0)
< 50	171 (19)	36 (15)	75 (20)	60 (20)
50–59	224 (24)	50 (20)	99 (27)	75 (25)
60–69	245 (27)	59 (24)	100 (27)	86 (29)
70–79	157 (17)	52 (21)	56 (15)	49 (16)
≥ 80	120 (13)	50 (20)	40 (11)	30 (10)
Mean ± standard deviation	63 ± 14	66 ± 15	61 ± 14	62 ± 13
Male	658 (71)	165 (66)	266 (72)	227 (76)
Race				
Missing ^b^	256 (28)	45(18)	87 (23)	124 (41)
Caucasian	550 (83)	165 (81)	238 (84)	147 (84)
African American	82 (12)	28 (14)	34 (12)	20 (11)
Asian	16 (2)	3 (1)	9 (3)	4 (2)
Other/unknown	17 (3)	8 (4)	4 (1)	5 (3)
Clinical presentation (may have more than one)				
Missing ^b^	134 (15)	45 (18)	87 (23)	2(1)
Prior MI	122 (15)	38 (19)	42 (15)	42 (14)
Prior CABG	50 (6)	22 (11)	15 (5)	13 (4)
CVD	41 (5)	14 (7)	10 (4)	17 (6)
Smoking	246 (31)	48 (24)	91 (32)	107 (36)
Hypertension	525 (67)	129 (63)	199 (70)	197 (66)
Dyslipidemia	424 (54)	92 (45)	168 (59)	164 (55)
Diabetes	189 (24)	59 (29)	57 (20)	73 (24)
Prior HF	41 (5)	20 (10)	13 (5)	8 (3)
BMI (kg/m^2^)				
Missing ^b^	162 (18)	71 (29)	90(24)	1(0)
Normal (< 25)	179 (24)	37 (21)	74 (26)	68 (23)
Overweight (25 ≤ BMI < 30)	288 (38)	67 (38)	95 (34)	126 (42)
Obesity (30 ≤ BMI < 35)	195 (26)	46 (26)	85 (30)	64 (21)
Severe Obesity (BMI ≥ 35)	97 (13)	28 (16)	28 (10)	41 (14)
Mean ± SD	29 ± 6	29 ± 6	29 ± 5	29 ± 6
Left ventricular ejection fraction				
Missing ^b^	374 (41)	249(100)	108 (29)	17 (6)
≤ 35%	104 (19)	–	50 (19)	54 (19)
36% - ≤45%	136 (25)	–	73 (28)	63 (22)
> 45%	307 (56)	–	141 (53)	166 (59)
Mean ± SD	47 ± 13	–	47 ± 12	49 ± 13

Data are N (%) or Mean ± SD. For any given characteristic, the denominator of percentage is all STEMIs with available data on that characteristic

^a^Ns are the number of STEMIs. There were a total of 921 STEMIs among 912 patients

^b^Denominator is all STEMIs (*N* = 921 for “All years”, *N* = 249 for “Before”, *N* = 372 for “During”, and *N* = 300 for “After”)

There were substantial decreases in the concentrations of almost all pollutants during the study period except for O_3_ (Table 2). From the BEFORE to the AFTER period, the median concentration of PM_2.5_ decreased by ~ 30%, AMP by ~ 40%, UFP by ~ 45%, BC and CO by ~ 50%, and SO_2_ by ~ 90%. However, the median O_3_ concentration increased by ~ 30% from the BEFORE period to the DURING and AFTER periods. Further description of these changes in Rochester and across New York State has been provided previously [1–3]. Pearson correlations between hourly pollutants concentrations are shown in Additional file 1: Table S1. PM_2.5_ was moderately correlated with AMP (r = 0.62–0.69), but poorly correlated with UFP (r = 0.12–0.22) in all years. AMP was moderately correlated with UFP in the BEFORE (r = 0.53) and DURING (r = 0.57) period, but the correlation was weaker in the AFTER period (*r* = 0.29).

**Table 2:** Distribution of hourly pollutant concentrations and weather characteristics (lag hour 0 of control periods) by period

					Percentile						
Pollutant	N	Mean	SD	IQR	Minimum	5th	25th	50th	75th	95th	Maximum
PM_2.5_ (μg/m^3^)											
All years (2005–2016)	2900	8.32	7.17	7.59	−7.85	0.19	3.63	6.83	11.22	21.86	76.15
Before (2005–2007)	788	10.37	8.74	8.40	−3.31	0.86	4.84	8.22	13.24	26.79	76.15
During (2008–2013)	1134	8.31	6.83	7.60	−3.94	0.25	3.80	6.75	11.40	21.87	47.15
After (2014–2016)	978	6.67	5.55	6.60	−7.85	−0.36	2.76	5.69	9.35	16.80	48.65
AMP (particles/cm^3^)											
All years (2005–2016)	2680	781	658	698	10	138	327	610	1025	1978	6578
Before (2005–2007)	566	1139	908	980	62	179	481	940	1461	2960	6015
During (2008–2013)	1156	739	596	672	32	140	317	586	988	1797	6578
After (2014–2016)	958	620	440	554	10	122	282	527	836	1469	2926
UFP (particles/cm^3^)											
All years (2005–2016)	2680	4286	3775	3702	70	738	1777	3232	5479	11,708	41,965
Before (2005–2007)	566	6916	5247	6046	325	1300	3235	5405	9281	16,720	41,965
During (2008–2013)	1156	3492	2832	3141	191	585	1466	2798	4606	8642	20,826
After (2014–2016)	958	3690	2958	2951	70	774	1728	2923	4679	9212	31,417
UFP(11-50 nm)(particles/cm^3^)											
All years (2005–2016)	2680	2963	2906	2755	30	392	1087	2106	3842	8588	32,747
Before (2005–2007)	566	4839	3958	4327	158	845	2015	3762	6342	12,708	32,747
During (2008–2013)	1156	2385	2148	2260	117	327	886	1770	3146	6515	17,182
After (2014–2016)	958	2552	2485	2182	30	419	1035	1805	3217	7202	29,559
UFP(50-100 nm)(particles/cm^3^)											
All years (2005–2016)	2680	1323	1242	1161	14	199	532	979	1693	3628	11,198
Before (2005–2007)	566	2077	1824	1701	73	316	878	1568	2579	5940	11,198
During (2008–2013)	1156	1107	972	963	48	174	470	842	1433	2943	8650
After (2014–2016)	958	1138	886	1016	14	185	504	905	1521	2851	6309
BC (μg/m^3^)											
All years (2005–2016)	3039	0.49	0.47	0.44	−0.07	0.06	0.19	0.36	0.63	1.36	5.31
Before (2005–2007)	783	0.69	0.60	0.63	−0.02	0.09	0.28	0.53	0.91	1.84	5.31
During (2008–2013)	1226	0.50	0.44	0.46	−0.07	0.08	0.21	0.37	0.66	1.34	3.74
After (2014–2016)	1030	0.33	0.28	0.30	−0.04	0.04	0.14	0.25	0.44	0.92	2.13
SO_2_ (ppb)											
All years (2005–2016)	3006	1.98	3.10	1.77	−0.09	0.09	0.32	1.00	2.09	7.00	68.00
Before (2005–2007)	822	4.11	4.41	3.00	0.00	1.00	2.00	3.00	5.00	11.00	68.00
During (2008–2013)	1179	1.54	2.20	1.70	−0.09	0.01	0.30	0.97	2.00	5.00	21.00
After (2014–2016)	1005	0.76	1.33	0.54	0.00	0.09	0.20	0.37	0.74	2.61	16.35
O_3_ (ppb)											
All years (2005–2016)	2992	26.6	14.1	18.0	0.0	2.0	17.0	26.0	35.0	50.0	83.0
Before (2005–2007)	828	21.9	14.6	21.0	0.0	1.0	10.0	21.0	31.0	49.0	77.0
During (2008–2013)	1162	28.1	14.0	16.0	0.0	4.0	20.0	28.0	36.0	51.0	83.0
After (2014–2016)	1002	28.9	12.9	17.0	0.0	6.0	20.0	29.0	37.0	50.0	72.0
CO (ppm)											
All years (2005–2016)	2939	0.30	0.20	0.23	−0.01	0.12	0.17	0.22	0.40	0.65	2.50
Before (2005–2007)	814	0.48	0.22	0.30	0.10	0.20	0.30	0.40	0.60	0.80	2.50
During (2008–2013)	1124	0.26	0.16	0.14	0.00	0.12	0.16	0.20	0.30	0.60	1.10
After (2014–2016)	1001	0.20	0.07	0.06	−0.01	0.12	0.16	0.19	0.22	0.33	1.02
Temperature (°F)											
All years (2005–2016)	3065	50.7	20.3	32.1	−5.0	19.2	34.8	50.0	66.9	83.7	96.4
Before (2005–2007)	839	48.4	18.9	30.5	1.4	20.2	33.2	46.9	63.7	78.7	95.9
During (2008–2013)	1196	49.5	20.2	32.1	4.8	20.1	33.4	46.6	65.5	84.9	96.4
After (2014–2016)	1030	53.9	21.0	32.9	−5.0	17.5	38.0	55.3	70.9	84.3	92.6
Relative Humidity											
All years (2005–2016)	3064	64.5	19.9	32.0	7.1	30.0	49.0	66.3	81.0	93.0	102.0
Before (2005–2007)	838	69.6	19.0	28.0	7.1	35.0	58.0	73.0	86.0	93.4	98.2
During (2008–2013)	1196	64.1	19.5	31.0	12.0	30.0	49.0	66.0	80.0	93.0	102.0
After (2014–2016)	1030	60.9	20.1	32.0	15.0	29.0	45.0	61.0	77.0	93.0	99.0

Number of total control periods = 3122; Number of control periods in the Before, During, and After period were 839, 1250, and 1033, respectively

*SD* standard deviation, *IQR* interquartile range

Over all periods, estimates of the relative rate of STEMI associated with increased PM_2.5_ and AMP concentrations were all close to 1.0 and not statistically significant (Table 3). However, increased rates of STEMI were associated with increased UFP and UFP 11-50 nm concentrations in the previous 1 (lag hour 0) and 3 h (lag hours 0–2). IQR increases in UFP concentrations in the previous 1 and 3 h were associated with 12% (95%CI: 3%, 22%) and 11% (95%CI: 2%, 22%) increases in the rate of STEMI, respectively. Similar to UFP, IQR increases in the particle number concentrations of UFP 11-50 nm (nucleation mode and spark ignition vehicle emissions [20–22]) at lag hour 0 and lag hours 0–2 were associated with 13% (95%CI: 4%, 22%) and 12% (95%CI: 3%, 21%) increases in the rate of STEMI, respectively. However, increases in UFP 50-100 nm (diesel vehicle emissions and residential wood burning; Aitken mode [20–22]) concentrations were not significantly associated with increases in the rate of STEMI, with effect estimates for all lag hours slightly greater than 1.0.

**Table 3:** Rate of STEMI associated with each interquartile range increase in pollutant concentration (2005–2016)

Lag hours	Interquartile range	No. of STEMI	Odds ratio	95% confidence interval
PM_2.5_(μg/m^3^)				
0	7.59	858	1.03	0.95, 1.13
0–2	7.33	848	1.05	0.96, 1.15
0–11	6.57	855	1.02	0.93, 1.12
0–23	6.21	854	1.01	0.91, 1.11
0–47	5.71	848	0.98	0.89, 1.09
0–71	5.30	833	0.96	0.87, 1.07
AMP (particles/cm^3^)				
0	698	779	1.07	0.97, 1.18
0–2	688	771	1.05	0.96, 1.16
0–11	693	780	1.03	0.91, 1.15
0–23	670	772	1.01	0.89, 1.14
0–47	602	763	1.01	0.88, 1.15
0–71	543	751	0.97	0.85, 1.11
UFP (particles/cm^3^)				
0	3702	779	1.12	1.03, 1.22
0–2	3506	771	1.11	1.02, 1.22
0–11	3265	780	1.05	0.93, 1.18
0–23	2955	772	1.00	0.88, 1.14
0–47	2568	763	1.06	0.93, 1.22
0–71	2349	751	1.00	0.86, 1.16
UFP 11-50 nm(particles/cm^3^)				
0	2755	779	1.13	1.04, 1.22
0–2	2551	771	1.12	1.03, 1.21
0–11	2344	780	1.03	0.92, 1.16
0–23	2151	772	0.97	0.85, 1.12
0–47	1862	763	1.04	0.90, 1.21
0–71	1682	751	0.98	0.84, 1.15
UFP 50-100 nm(particles/cm^3^)				
0	1161	779	1.06	0.97, 1.14
0–2	1108	771	1.06	0.97, 1.15
0–11	1118	780	1.07	0.96, 1.20
0–23	1035	772	1.05	0.94, 1.19
0–47	952	763	1.09	0.95, 1.25
0–71	844	751	1.02	0.89, 1.18
Black Carbon (μg/m^3^)				
0	0.44	891	1.09	1.01, 1.18
0–2	0.43	885	1.09	1.00, 1.18
0–11	0.42	891	1.05	0.95, 1.16
0–23	0.39	893	1.05	0.94, 1.16
0–47	0.35	884	1.10	0.98, 1.23
0–71	0.33	881	1.03	0.91, 1.17
SO_2_(ppb)				
0	1.77	889	1.04	0.99, 1.09
0–2	2.20	884	1.09	1.02, 1.18
0–11	2.27	889	1.17	1.06, 1.29
0–23	2.30	896	1.15	1.01, 1.30
0–47	2.19	886	1.14	0.98, 1.33
0–71	2.22	883	1.18	0.99, 1.41
O_3_ (ppb)				
0	18.0	884	0.89	0.77, 1.03
0–2	17.3	872	0.90	0.78, 1.04
0–11	15.2	888	0.92	0.80, 1.06
0–23	13.7	893	0.92	0.79, 1.07
0–47	12.9	887	0.87	0.73, 1.03
0–71	12.3	883	0.90	0.75, 1.07
CO (ppm)				
0	0.23	866	1.15	1.02, 1.29
0–2	0.23	852	1.13	1.00, 1.28
0–11	0.21	873	1.10	0.96, 1.27
0–23	0.22	875	1.07	0.91, 1.25
0–47	0.21	870	1.10	0.94, 1.30
0–71	0.21	864	1.08	0.91, 1.29

Odds ratios were estimated from conditional logistic regression models adjusting for mean temperature and relative humidity during the same lag hour(s)

*STEMI* ST-elevation myocardial infarction

IQR increases in BC concentrations (lag hour 0: 9%, 95%CI: 1%, 18%; lag hours 0–2: 9%, 95%CI: 0%, 18%) and CO concentrations (lag hour 0: 15%, 95%CI: 2%, 29%; lag hours 0–2: 13%, 95%CI: 0%, 28%) had similar patterns as UFP. Increased SO_2_ concentrations were associated with increased rates of STEMI in the previous 3 to 72 h, with statistically significant increases in the previous 3 to 24 h. Relative rates ranged from 1.14 to 1.18 in the previous 12 to 72 h. The rates of STEMI associated with each IQR increase in O_3_ concentrations in the previous 1 to 72 h were all less than 1.0 (OR = 0.87–0.92), but none were statistically significant. These results were essentially the same after additional adjustment for holiday (Additional file 1: Table S2). The rate of STEMI associated with increased UFP concentrations in the previous hour was not substantially different by age, sex, smoking, diabetes, dyslipidemia, and heart failure (Additional file 1: Table S3), although the relative rate was larger in males than females (1.16, 95%CI: 1.05–1.29 in males vs. 1.04, 95%CI: 0.89–1.21 in females). Further, there was no evidence of a non-linear UFP concentration-response function, as the rate of STEMI associated with increased UFP in the previous hour (linear function, 1 df) had the lowest AIC (2168.9) compared to models using a natural spline with 2 df (AIC = 2170.7), 3 df (2171.9), 4 df (AIC = 2173.7), or 5 df (AIC = 2174.9).

**Fig. 1:**
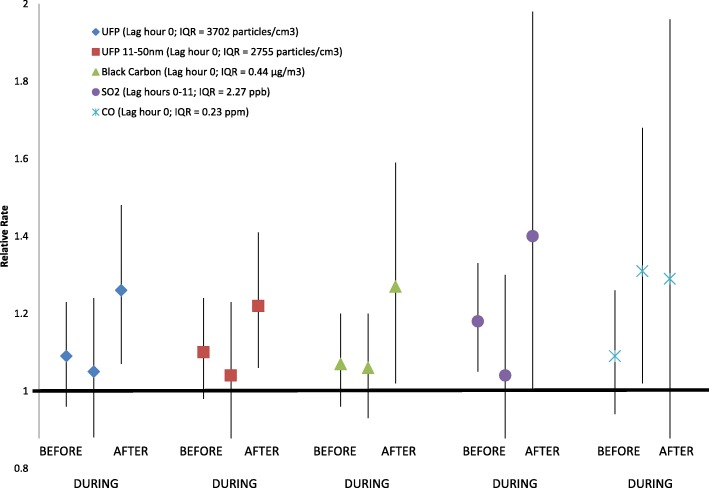
Rate of STEMI associated with each interquartile range increase in pollutant concentration, by time period. For each pollutant, the lag hour with the largest main effect that was statistically significant was selected for presentation. Error bars are 95% confidence intervals

Increases in the rate of STEMI associated with increased concentrations of UFP, UFP 11-50 nm, BC, and SO_2_ were larger in the AFTER period compared to those in the BEFORE and DURING periods (Fig. 1; Table 4). In the previous hour (lag hour with the largest UFP relative rate in Table 3), each IQR (3702 particles/cm^3^) increase in UFP concentration was associated with 9% (95%CI: − 4%, 23%), 5% (95%CI: − 12%, 24%), and 26% (95%CI: 7%, 48%) increases in the rate of STEMI, in the BEFORE, DURING, and AFTER periods, respectively. This pattern remained even when separately including interaction terms between air pollution and age, sex, smoking, diabetes, dyslipidemia, and heart failure in the model (Additional file 1: Table S4). There were similar patterns for UFP in the previous 3 h, UFP 11-50 nm in the previous hour and previous 3 h, BC in the previous hour, and SO_2_ in the previous 1 to 24 h. However, the pattern for CO relative rates was different. Each IQR (0.23 ppm) increase in CO concentration at lag hour 0 was associated with 9% (− 6 to 26%), 31% (2 to 68%), and 29% (− 15 to 96%) increases in the rate of STEMI, in the BEFORE, DURING, and AFTER periods, respectively, showing similarly larger relative rates in the DURING and AFTER periods. We did not observe a pattern of effect modification by period for the associations between STEMI and PM_2.5_, UFP 50-100 nm, AMP, or O_3_.

**Table 4:** Rate of STEMI associated with each interquartile range increase in pollutant concentration in the Before, During, and After periods

Lag hours	IQR	Before (2005–2007)			During (2008–2013)			After (2014–2016)		
		No. of STEMI	OR	95%CI	No. of STEMI	OR	95%CI	No. of STEMI	OR	95%CI
PM_2.5_ (μg/m^3^)										
0	7.59	234	1.03	0.91, 1.17	339	1.07	0.92, 1.24	285	0.99	0.81, 1.21
0–2	7.33	233	1.04	0.92, 1.18	333	1.04	0.90, 1.21	282	1.08	0.86, 1.34
0–11	6.57	235	1.03	0.91, 1.15	336	1.01	0.87, 1.18	284	1.00	0.81, 1.24
0–23	6.21	234	1.00	0.89, 1.13	335	1.00	0.85, 1.17	285	1.03	0.83, 1.28
0–47	5.71	235	0.96	0.84, 1.09	328	0.98	0.83, 1.17	285	1.07	0.85, 1.34
0–71	5.30	232	0.91	0.79, 1.05	321	0.98	0.82, 1.18	280	1.11	0.88, 1.41
AMP (particles/cm^3^)										
0	698	166	1.07	0.93, 1.23	328	1.07	0.93, 1.24	285	1.08	0.84, 1.38
0–2	688	165	1.07	0.93, 1.23	322	1.03	0.89, 1.20	284	1.05	0.82, 1.35
0–11	693	165	0.99	0.84, 1.18	328	1.08	0.90, 1.29	287	0.99	0.75, 1.29
0–23	670	161	0.95	0.79, 1.14	327	1.05	0.87, 1.27	284	1.06	0.80, 1.39
0–47	602	156	0.98	0.81, 1.19	324	1.01	0.83, 1.23	283	1.06	0.80, 1.42
0–71	543	148	0.95	0.78, 1.16	321	0.97	0.80, 1.18	282	1.00	0.75, 1.34
UFP (particles/cm^3^)										
0	3702	166	1.09	0.96, 1.23	328	1.05	0.88, 1.24	285	1.26	1.07, 1.48
0–2	3506	165	1.12	0.99, 1.27	322	0.99	0.83, 1.18	284	1.24	1.04, 1.48
0–11	3265	165	1.11	0.94, 1.32	328	0.97	0.79, 1.21	287	1.04	0.81, 1.32
0–23	2955	161	1.00	0.83, 1.21	327	0.96	0.77, 1.20	284	1.07	0.83, 1.38
0–47	2568	156	1.06	0.86, 1.30	324	1.01	0.80, 1.28	283	1.14	0.87, 1.49
0–71	2349	148	0.95	0.76, 1.18	321	0.94	0.73, 1.21	282	1.18	0.89, 1.56
UFP 11-50 nm (particle/cm^3^)										
0	2755	166	1.10	0.98, 1.24	328	1.04	0.87, 1.23	285	1.22	1.06, 1.41
0–2	2551	165	1.12	1.00, 1.27	322	0.98	0.82, 1.17	284	1.22	1.04, 1.42
0–11	2344	165	1.10	0.94, 1.31	328	0.93	0.75, 1.16	287	1.02	0.81, 1.29
0–23	2151	161	1.01	0.83, 1.23	327	0.90	0.71, 1.14	284	1.01	0.78, 1.31
0–47	1862	156	1.09	0.87, 1.35	324	0.98	0.76, 1.26	283	1.05	0.79, 1.39
0–71	1682	148	0.95	0.75, 1.19	321	0.91	0.69, 1.20	282	1.13	0.84, 1.51
UFP 50-100 nm (particles/cm^3^)										
0	1161	166	1.03	0.92, 1.16	328	1.04	0.90, 1.21	285	1.14	0.95, 1.37
0–2	1108	165	1.07	0.95, 1.21	322	1.01	0.86, 1.18	284	1.12	0.92, 1.35
0–11	1118	165	1.09	0.92, 1.30	328	1.06	0.87, 1.29	287	1.04	0.84, 1.29
0–23	1035	161	0.99	0.83, 1.19	327	1.06	0.87, 1.30	284	1.15	0.91, 1.45
0–47	952	156	1.01	0.82, 1.25	324	1.07	0.85, 1.34	283	1.27	0.98, 1.64
0–71	844	148	0.95	0.76, 1.17	321	0.98	0.78, 1.24	282	1.22	0.93, 1.59
BC (μg/m^3^)										
0	0.44	241	1.07	0.96, 1.20	353	1.06	0.93, 1.20	297	1.27	1.02, 1.59
0–2	0.43	241	1.08	0.97, 1.21	347	1.07	0.94, 1.23	297	1.16	0.92, 1.47
0–11	0.42	241	1.04	0.90, 1.19	352	1.05	0.90, 1.24	298	1.09	0.84, 1.42
0–23	0.39	241	1.01	0.87, 1.17	353	1.05	0.89, 1.24	299	1.20	0.92, 1.56
0–47	0.35	236	1.06	0.91, 1.24	349	1.08	0.90, 1.30	299	1.30	0.98, 1.73
0–71	0.33	233	1.00	0.84, 1.18	348	0.98	0.80, 1.21	300	1.32	0.98, 1.78
SO_2_ (ppb)										
0	1.77	243	1.02	0.97, 1.08	353	1.02	0.91, 1.14	293	1.18	1.01, 1.36
0–2	2.20	243	1.07	0.98, 1.17	348	1.06	0.90, 1.26	293	1.34	1.07, 1.69
0–11	2.27	242	1.18	1.05, 1.33	351	1.04	0.84, 1.30	296	1.40	1.00, 1.98
0–23	2.30	245	1.14	0.99, 1.32	354	0.98	0.73, 1.31	297	1.62	1.10, 2.38
0–47	2.19	244	1.11	0.93, 1.32	346	1.08	0.77, 1.53	296	1.61	0.98, 2.65
0–71	2.22	242	1.10	0.89, 1.36	344	1.37	0.92, 2.05	297	1.55	0.84, 2.83
O_3_ (ppb)										
0	18.0	245	0.88	0.70, 1.11	350	0.91	0.74, 1.13	289	0.88	0.68, 1.13
0–2	17.3	244	0.86	0.68, 1.08	342	0.94	0.76, 1.16	286	0.91	0.70, 1.17
0–11	15.2	243	0.87	0.69, 1.09	349	0.98	0.79, 1.21	296	0.91	0.71, 1.17
0–23	13.7	247	0.91	0.70, 1.16	350	0.96	0.76, 1.20	296	0.89	0.68, 1.16
0–47	12.9	247	0.75	0.57, 0.99	344	0.96	0.74, 1.25	296	0.90	0.67, 1.21
0–71	12.3	246	0.78	0.59, 1.04	340	1.06	0.80, 1.40	297	0.86	0.63, 1.18
CO (ppm)										
0	0.23	242	1.09	0.94, 1.26	334	1.31	1.02, 1.68	290	1.29	0.85, 1.96
0–2	0.23	242	1.09	0.94, 1.26	324	1.28	1.00, 1.65	286	1.11	0.69, 1.78
0–11	0.21	241	1.05	0.88, 1.25	337	1.29	0.97, 1.70	295	1.05	0.64, 1.70
0–23	0.22	243	0.99	0.81, 1.20	335	1.26	0.93, 1.71	297	1.20	0.70, 2.06
0–47	0.21	242	1.03	0.84, 1.26	332	1.32	0.96, 1.82	296	1.14	0.62, 2.11
0–71	0.21	242	1.00	0.81, 1.24	326	1.35	0.95, 1.93	296	1.13	0.56, 2.27

Odds ratios were estimated from conditional logistic regression models adjusting for mean temperature and relative humidity during the same lag hour(s)

*IQR* interquartile range, *STEMI* ST-elevation myocardial infarction, *OR* odds ratio, *95%CI* 95% confidence interval

## Discussion

In Rochester, NY from 2005 to 2016, increased rates of STEMI were associated with increased concentrations of UFP, UFP 11-50 nm, BC, and CO in the previous 1 to 3 h, as well as increased SO_2_ concentration in the previous 3 to 72 h. Further, we found larger rates of STEMI associated with UFP, UFP11-50 nm, BC, and SO_2_ in the period after air quality policy implementation and economic changes (AFTER period: 2014–2016) compared to the periods BEFORE (2005–2007) or DURING (2008–2013) these changes. Given concomitant changes in pollutant composition over the three periods, this difference in relative rate by period may reflect differential triggering of STEMI by specific PM constituents.

In our previous studies, Evans et al. reported a 17% increase in the rate of STEMI associated with each 7.1 μ g/m^3^ increase in PM_2.5_ concentrations in the previous hour using STEMI from 2007 to 2012 and subjects living in Monroe County, NY [11], and Gardner et al. reported a 18% increased rate associated with each 7.1 μg/m^3^ increase in PM _2.5_ in the previous hour using STEMI from 2007 to 2010 and subjects living within 15 miles of the Rochester monitoring station [12]. When using data from 2005 to 2016, but the same analysis methods for subjects living within 15 miles of the monitoring station, we found only a 3% (− 5 to 12%) increased rate of STEMI associated with each 7.1 μg/m^3^ increase in PM_2.5_ in the previous hour. Differences in PM composition between the study periods and differences in study populations may explain, in part, this discrepancy. Most, but not all other case-crossover studies also reported increased risks/odds/rates of STEMI associated with short-term increases in PM_2.5_ concentrations [13–17, 27]. However, most of these studies did not have symptom onset time and thus were not able to examine triggering of MI by increased pollutant concentrations in the previous few hours, perhaps missing an etiologically important time window. Most of these studies observed the largest increased rate of STEMI associated with pollutant concentrations in the concurrent or previous day, with excess relative rate estimates (associated with each 10 μg/m^3^ increase in PM_2.5_ concentration) ranging from 5 to 15% [13, 14, 16, 17].

In contrast with the lack of findings for PM_2.5_, we found a 12% increase in the rate of STEMI associated with each 3702 particles/cm^3^ increase in UFP concentration in the previous hour. Analyses of different UFP size fractions (11-50 nm and 50-100 nm) suggested that this observed increase in STEMI rate associated with UFPs was primarily driven by UFP 11-50 nm, which are mostly gasoline emissions and nucleation mode particles [20, 22]. UFPs comprise a large particle number concentration, but contribute little mass to PM_2.5_ [28, 29]. In the present study, PM_2.5_ mass concentration and UFP particle number concentration were poorly correlated (*r* = 0.21). Both UFPs and PM_2.5_ have been associated with adverse cardiovascular responses including systematic inflammation, oxidative stress, endothelial dysfunction, thrombosis and coagulation, and autonomic dysfunction [7, 10, 30, 31]. However, compared to larger particle fractions (e.g. AMP), UFPs deposit more deeply into the lung and may even escape clearance mechanisms and translocate into the circulatory system [32, 33]. Therefore, UFPs may exert adverse health effects via pathophysiological pathways different from PM_2.5_ and AMP [30].

Consistent with a meta-analysis of air pollutants and MI by Mustafic et al. [8], we observed increased rates of STEMI associated with increased CO and SO_2_ concentrations, but not O_3_. Our findings of an increased rate of STEMI associated with increased concentrations of BC in the previous hour (Excess Rate = 9%, 95% CI: 1%, 18%; per 0.44 μg/m^3^) and previous 3 h (Excess Rate = 9%, 95% CI: 0%, 18%; per 0.43 μg/m^3^) are generally consistent with a study by Zanobetti and Schwartz that reported an 8.3% (95% CI: 0.2%, 15.8%) increase in the risk of hospitalization for MI associated with each 1.7 μg/m^3^ increase in BC concentrations in the previous 2 days in Boston, MA from 1995 to 1999 [34].

An interesting finding of our study is the pattern of larger rates of STEMI associated with increased concentrations of UFP, UFP 11-50 nm, BC, and SO_2_ in the period AFTER pollutant source and economic changes compared to the periods BEFORE or DURING these changes, which is contradictory to our a priori hypothesis. However, this is consistent with our other work conducted in New York State using hospital admissions of NY residents living near PM_2.5_ monitoring stations in Buffalo, Albany, Rochester, and New York City during the same study period of 2005–2016 [35]. In that analysis, we observed a greater excess rate of ischemic heart disease hospital admissions associated with increased PM_2.5_ concentrations in the previous 2 days in the AFTER period (2.8%; 2014–2016) than in the DURING (0.6%; 2008–2013) or BEFORE (0.8%; 2005–2007) periods [35]. Our finding of a greater excess rate of STEMI associated with increased UFP concentrations in the previous hour in the AFTER period (26%) compared to the DURING (5%) and BEFORE (9%) periods, is similar. Together, these findings may reflect the same change in PM composition and differential triggering of acute cardiovascular events by one or more PM components and/or PM mixtures. We suspect that secondary organic compounds and particle-bound reactive oxygen species (ROS) may play a role in the stronger adverse effect of these pollutants (i.e., UFP, UFP 11-50 nm, BC, SO_2_,) after economic changes and policy initiatives that reduced SO_2_ and NOx emissions. It is also possible that this lower relative rate estimated in the AFTER period was attributable to non-linearity of the exposure-response function. However, this seems unlikely, since our sensitivity analyses of the rate of STEMI associated with UFP in the previous hour suggested a linear (1 df) exposure-response function fit the data best.

Concentrations of sulfate and nitrate have decreased from 2005 to 2016 across Rochester and New York State following air quality policy initiatives and economic changes [1, 36]. However, Squizzato et al. and Zhang et al. reported that over this same period, secondary organic carbon increased across New York State [35, 37]. These decreases in SO_2_ and NOx emissions may have led to an increased oxidation rate of volatile organic compounds by hydroxyl radicals that would have otherwise reacted with the pollutant gases [37, 38]. The oxidation of SO_2_ and NO_2_ forms strong acid, but oxidation of organic compounds forms secondary organic aerosol and produces ROS such as peroxy radicals and peroxide compounds [39]. ROS along with the secondary organics can deposit on particles (particle-bound ROS), which can then be inhaled leading to oxidative stress [40]. Oxidative stress and associated inflammation are hypothesized to be key mechanisms underlying the adverse cardiovascular health effect associated with air pollutants [7]. Although it is well known that ROS can be formed in situ after particles are deposited in the respiratory tract, little attention has been focused on particle-bound ROS [39, 40]. We suspect that PM high in secondary organics and ROS may be more toxic than PM high in secondary sulfate and nitrate in triggering MI.

Furthermore, recent source apportionment analyses across New York State including Rochester suggest that although PM_2.5_ concentrations and contributions from most major source types were decreasing, spark-ignition vehicle emissions were increasing [4, 37]. Rich et al. (2019) found increased rates of cardiovascular hospitalizations associated with increased concentrations of spark ignition vehicles and diesel emissions in the previous few days [41]. The correlation coefficient between secondary organic carbon and the spark-ignition vehicle contribution to PM_2.5_ mass in Rochester was 0.73, suggesting that the increased formation of secondary organic carbon in the AFTER period may be associated with spark-ignition automotive emissions. This result is consistent with our findings that the UFP 11–50 nm fraction (marker for nucleation and spark ignition vehicle emissions [23]) was the primary contributor for the increased rate of STEMI associated with UFP. Diesel emissions also contribute to nucleation mode particles [23]. UFP 50–100 nm fraction (a larger mode indicator of diesel vehicle emissions) was not associated with increased rates of STEMI. Taken together, the observed stronger effect of UFP and BC on the rate of STEMI in the AFTER period may be explained by increased formation of secondary organics and ROS in the atmosphere, in part from spark-ignition vehicle emissions, and the subsequent increase in particle-bound ROS. These findings suggest that although the concentrations of PM have decreased following policy initiatives and economic changes, changing composition of PM may make the same dose of PM (e.g. each 3702 particles/cm^3^ of UFP) more toxic in triggering STEMI.

SO_2_ also had a stronger effect in the AFTER period, which may represent the influence of the remaining emissions from diesel traffic near the monitoring site including heavy-duty diesel traffic on the major roads and the adjacent diesel trains. Although on-road diesel fuel went to ultralow sulfur in 2006 and non-road diesel sold in NYS went to ultralow in 2012, out-of-state non-road diesel such as that used in diesel railroad engines was not ultralow sulfur until 2014. There would also be an influence from upwind coal-fired power plants in Dunkirk, NY (shutdown December 31, 2015) and Tonawanda, NY (Huntley Generating Station closed March 1, 2016). Thus, SO_2_ may be a surrogate for fresh secondary organic aerosol being produced in the upwind domain and transported with the SO_2_. CO had a stronger influence in the DURING and AFTER periods. CO could be a surrogate for spark-ignitions vehicles and residential wood combustion. Squizzato et al. [37] reported that car registrations increased in Monroe County after 2010. However, this also may be a result of a more sensitive CO monitor starting in 2011, resulting in reduced exposure misclassification and less downward bias, and larger relative rates in the DURING and AFTER periods. Health effect studies based on apportioned source contributions are needed to further assess these associations.

This study had several strengths, including a large sample size and multiple years of exposure data, resulting in increased statistical power. Additionally, we used symptom onset time, estimated by the patient and treating physician, to define the start time of each STEMI event, likely resulting in less exposure error than previous studies that used only hospital arrival date/time [8, 13–17, 27]. Further, it allowed us to examine whether the rate of STEMI increased a few hours after increases in ambient pollutant concentrations, rather than just on or after one or more days.

However, our study also had several limitations. First, we used air pollution measurements from a central monitoring station to assign exposure to all subjects living within a 15-mile radius of the site, regardless of the specific distance from the monitoring station to subject’s residence and/or where they worked and spent time. However, this exposure error is likely to be non-differential with regard to case and control periods and thus, should result only in underestimates of the relative rate of STEMI. The magnitude of this measurement error and underestimation may vary for different pollutants. UFP is thought to be more spatially variable than PM_2.5_ and AMP, and our previous work observed high spatial heterogeneity in BC across the Rochester area [42]. Although UFP and BC may be subject to greater exposure error, we observed increased rates of STEMI associated with these two pollutants. The true effect estimates in the absence of exposure measurement error might be even larger.

Second, the three time periods BEFORE (2005–2007), DURING (2008–2013), and AFTER (2014–2016) were selected based on times of emission changes. However, for many of the policies and emission changes, there are no well-defined time windows for the change because the interventions were implemented over time. For example, emission standards are only for new vehicles, but we do not know how quickly people buy new cars and trucks and take old vehicles out of service. We determined the best approach was to select groups of years where there were moderately constant or specifically changing emissions. As is shown in Additional file 1: Figure S1 from Squizzato et al. [2], SO_2_ emissions from coal-fired power plants in western New York were relatively high before 2008, and since 2008 it dropped substantially and remained at a relatively low level after 2013. The differences in air pollution concentrations and compositions among the 3 periods were analyzed and reported in Masiol et al. [4], Zhang et al. [35], and Croft et al. [43]. In the present study, the BEFORE, DURING, and AFTER period is only a proxy for the time of air quality policy implementation/intervention or economic change.

Third, patients in the three periods were perhaps different regarding several clinical characteristics associated with an increased rate of MI [44]. STEMI patients included in the BEFORE period were older and had a higher prevalence of diabetes and heart failure than patients in the AFTER period. By contrast, STEMI patients in the AFTER period were more often male, smokers, and more often had dyslipidemia. Although these characteristics may be related to susceptibility to acute effects of short-term air pollution exposure, our sensitivity analyses show that the pattern of larger increased rate of STEMI associated with increased UFP concentrations at lag hour 0 in the AFTER period compared to the BEFORE and DURING periods remained when also adjusting for interactions between air pollutant and age, sex, smoking, diabetes, dyslipidemia, and heart failure in separate models (Additional file 1: Table S4), suggesting these differences in period-specific effect estimates were not likely due to differences in the characteristics of the STEMI patients by period and interactions between those characteristics and air pollutant concentrations. Furthermore, as discussed above, the pattern of a larger increased rate of STEMI associated with increased UFP in the AFTER period in the present study is similar to the larger rate of ischemic heart disease hospital admissions associated with increased PM_2.5_ in the AFTER period reported by Zhang et al. across New York State [35]. This correspondence suggests that difference in study population by period may not be the sole reason for our observed period-specific effects. It is also of note that since the proportion of missing data on clinical characteristics was large in the BEFORE period (18% missing), we may not have accurately compared STEMI patient characteristics between periods.

Fourth, we did not have data on influenza in our study subjects or influenza epidemics occurring during the study period, and thus did not adjust for this variable in our analyses. However, influenza may have been a mediator of the association between air pollution and STEMI, rather than a confounder, as several studies have reported triggering of respiratory infection by short term increases in air pollutant concentrations [43, 45–48], and others have suggested that influenza may trigger acute myocardial infarction [49–51]. Finally, although our inference was primarily made by considering the pattern of STEMI response to pollutants across multiple lag times and not just whether each was statistically significant, type I error in the assessment of the main effect of air pollution on STEMI might be inflated due to multiple comparisons.

## Conclusions

In summary, increased rates of STEMI associated with increased UFP, UFP-11-50 nm, BC, and SO_2_ concentrations were higher in the AFTER period (2014–2016) compared to the BEFORE (2005–2007) and DURING (2008–2013) periods. This may be due to changes in PM composition (i.e. higher secondary organic carbon and particle-bound ROS) following multiple air quality policies and economic changes. The sources and the role of secondary organics and particle-bound ROS in triggering MI need to be further studied.

## Additional file

Additional file 1: Supplementary materials. (DOCX 393 kb)

## Data Availability

The datasets generated and/or analyzed during the current study are not publicly available since it contains patient health information.

## Abbreviations

AMP: Accumulation mode particle
BC: Black carbon
CABG: Coronary Artery Bypass Grafting
Cath Lab: Catheterization Laboratory
CO: Carbon monoxide
IQR: Interquartile range
NOx: Nitrogen oxide
O_3_: Ozone
OR: Odds ratio
PM: Particulate matter
SO_2_: Sulfur dioxide
STEMI: ST elevation myocardial infarctions
UFP: Ultrafine particle
URMC: University of Rochester Medical Center

## References

[1] Emami F, Masiol M, Hopke PK (2018). Air pollution at Rochester, NY: long-term trends and multivariate analysis of upwind SO2 source impacts. Sci Total Environ.

[2] Squizzato S, Masiol M, Rich DQ, Hopke PK (2018). PM2.5 and gaseous pollutants in New York State during 2005–2016: Spatial variability, temporal trends, and economic influences. Atmos Environ.

[3] Masiol M, Squizzato S, Chalupa DC, Utell MJ, Rich DQ, Hopke PK (2018). Long-term trends in submicron particle concentrations in a metropolitan area of the northeastern United States. Sci Total Environ.

[4] Masiol M, Squizzato S, Rich DQ, Hopke PK (2019). Long-term trends (2005–2016) of source apportioned PM2.5 across New York state. Atmos Environ.

[5] Bourdrel T, Bind M-A, Béjot Y, Morel O, Argacha J-F (2017). Cardiovascular effects of air pollution. Arch Cardiovasc Dis.

[6] Brook RD (2008). Cardiovascular effects of air pollution. Clin Sci (London, England : 1979).

[7] Brook RD, Rajagopalan S, Pope CA, Brook JR, Bhatnagar A, Diez-Roux AV, Holguin F, Hong Y, Luepker RV, Mittleman MA (2010). Particulate matter air pollution and cardiovascular disease: an update to the scientific statement from the American Heart Association. Circulation.

[8] Mustafic H, Jabre P, Caussin C, Murad MH, Escolano S, Tafflet M, Perier MC, Marijon E, Vernerey D, Empana JP (2012). Main air pollutants and myocardial infarction: a systematic review and meta-analysis. JAMA.

[9] Rich DQ, Kipen HM, Huang W, Wang G, Wang Y, Zhu P, Ohman-Strickland P, Hu M, Philipp C, Diehl SR (2012). Association between changes in air pollution levels during the Beijing Olympics and biomarkers of inflammation and thrombosis in healthy young adults. Jama.

[10] Rich DQ, Zareba W, Beckett W, Hopke PK, Oakes D, Frampton MW, Bisognano J, Chalupa D, Bausch J, O'Shea K (2012). Are ambient ultrafine, accumulation mode, and fine particles associated with adverse cardiac responses in patients undergoing cardiac rehabilitation?. Environ Health Perspect.

[11] Evans KA, Hopke PK, Utell MJ, Kane C, Thurston SW, Ling FS, Chalupa D, Rich DQ (2017). Triggering of ST-elevation myocardial infarction by ambient wood smoke and other particulate and gaseous pollutants. J Expo Sci Environ Epidemiol.

[12] Gardner B, Ling F, Hopke PK, Frampton MW, Utell MJ, Zareba W, Cameron SJ, Chalupa D, Kane C, Kulandhaisamy S (2014). Ambient fine particulate air pollution triggers ST-elevation myocardial infarction, but not non-ST elevation myocardial infarction: a case-crossover study. Part Fibre Toxicol.

[13] Akbarzadeh MA, Khaheshi I, Sharifi A, Yousefi N, Naderian M, Namazi MH, Safi M, Vakili H, Saadat H, Alipour Parsa S (2018). The association between exposure to air pollutants including PM10, PM2.5, ozone, carbon monoxide, sulfur dioxide, and nitrogen dioxide concentration and the relative risk of developing STEMI: a case-crossover design. Environ Res.

[14] Argacha JF, Collart P, Wauters A, Kayaert P, Lochy S, Schoors D, Sonck J, de Vos T, Forton M, Brasseur O (2016). Air pollution and ST-elevation myocardial infarction: a case-crossover study of the Belgian STEMI registry 2009-2013. Int J Cardiol.

[15] Liu H, Tian Y, Cao Y, Song J, Huang C, Xiang X, Li M, Hu Y (2018). Fine particulate air pollution and hospital admissions and readmissions for acute myocardial infarction in 26 Chinese cities. Chemosphere.

[16] Pope CA, Muhlestein JB, Anderson JL, Cannon JB, Hales NM, Meredith KG, Le V, Horne BD. Short-Term Exposure to Fine Particulate Matter Air Pollution Is Preferentially Associated With the Risk of ST-Segment Elevation Acute Coronary Events. J Am Heart Assoc. 2015;4(12):e002506. 10.1161/JAHA.115.002506 PMC4845284 26645834

[17] Zhang Q, Qi W, Yao W, Wang M, Chen Y, Zhou Y (2016). Ambient particulate matter (PM2.5/PM10) exposure and emergency department visits for acute myocardial infarction in Chaoyang District, Beijing, China during 2014: A case-crossover study. J Epidemiol.

[18] Rich David Q., Özkaynak Halûk, Crooks James, Baxter Lisa, Burke Janet, Ohman-Strickland Pamela, Thevenet-Morrison Kelly, Kipen Howard M., Zhang Junfeng, Kostis John B., Lunden Melissa, Hodas Natasha, Turpin Barbara J. (2013). The Triggering of Myocardial Infarction by Fine Particles Is Enhanced When Particles Are Enriched in Secondary Species. Environmental Science & Technology.

[19] O'Gara PT, Kushner FG, Ascheim DD, Casey DE, Chung MK, de Lemos JA, Ettinger SM, Fang JC, Fesmire FM, Franklin BA (2013). 2013 ACCF/AHA guideline for the management of ST-elevation myocardial infarction: a report of the American College of Cardiology Foundation/American Heart Association task force on practice guidelines. Circulation.

[20] Kittelson DB, Watts WF, Johnson JP, Schauer JJ, Lawson DR (2006). On-road and laboratory evaluation of combustion aerosols—Part 2:: Summary of spark ignition engine results. J Aerosol Sci.

[21] Kittelson DB, Watts WF, Johnson JP (2006). On-road and laboratory evaluation of combustion aerosols—Part1: summary of diesel engine results. J Aerosol Sci.

[22] Kasumba J, Hopke PK, Chalupa DC, Utell MJ (2009). Comparison of sources of submicron particle number concentrations measured at two sites in Rochester, NY. Sci Total Environ.

[23] Kittelson DB (1998). Engines and nanoparticles: a review. J Aerosol Sci.

[24] Masiol M, Squizzato S, Cheng M-D, Rich DQ, Hopke PK (2019). Differential probability functions for investigating long-term changes in local and regional air pollution sources. Aerosol Air Qual Res.

[25] Levy D, Lumley T, Sheppard L, Kaufman J, Checkoway H (2001). Referent selection in case-crossover analyses of acute health effects of air pollution. Epidemiology.

[26] Maclure M (1991). The case-crossover design: a method for studying transient effects on the risk of acute events. Am J Epidemiol.

[27] Butland BK, Atkinson RW, Milojevic A, Heal MR, Doherty RM, Armstrong BG, MacKenzie IA, Vieno M, Lin C, Wilkinson P (2016). Myocardial infarction, ST-elevation and non-ST-elevation myocardial infarction and modelled daily pollution concentrations: a case-crossover analysis of MINAP data. Open Heart.

[28] Jeong C-H, Hopke PK, Chalupa D, Utell M (2004). Characteristics of nucleation and growth events of ultrafine particles measured in Rochester, NY. Environ Sci Technol.

[29] Oberdorster G, Oberdorster E, Oberdorster J (2005). Nanotoxicology: an emerging discipline evolving from studies of ultrafine particles. Environ Health Perspect.

[30] Gong J, Zhu T, Kipen H, Wang G, Hu M, Guo Q, Ohman-Strickland P, Lu SE, Wang Y, Zhu P (2014). Comparisons of ultrafine and fine particles in their associations with biomarkers reflecting physiological pathways. Environ Sci Technol.

[31] Wang M, Utell MJ, Schneider A, Zareba W, Frampton MW, Oakes D, Hopke PK, Wiltshire J, Kane C, Peters A (2016). Does total antioxidant capacity modify adverse cardiac responses associated with ambient ultrafine, accumulation mode, and fine particles in patients undergoing cardiac rehabilitation?. Environ Res.

[32] Geiser M, Rothen-Rutishauser B, Kapp N, Schurch S, Kreyling W, Schulz H, Semmler M, Im Hof V, Heyder J, Gehr P (2005). Ultrafine particles cross cellular membranes by nonphagocytic mechanisms in lungs and in cultured cells. Environ Health Perspect.

[33] Moller W, Felten K, Sommerer K, Scheuch G, Meyer G, Meyer P, Haussinger K, Kreyling WG (2008). Deposition, retention, and translocation of ultrafine particles from the central airways and lung periphery. Am J Respir Crit Care Med.

[34] Zanobetti A, Schwartz J (2006). Air pollution and emergency admissions in Boston, MA. J Epidemiol Community Health.

[35] Zhang W, Lin S, Hopke PK, Thurston SW, van Wijngaarden E, Croft D, Squizzato S, Masiol M, Rich DQ (2018). Triggering of cardiovascular hospital admissions by fine particle concentrations in New York state: before, during, and after implementation of multiple environmental policies and a recession. Environ Pollut.

[36] Rattigan OV, Civerolo KL, Felton HD, Schwab JJ, Demerjian KL (2016). Long term trends in New York: PM2.5 mass and particle components. Aerosol Air Qual Res.

[37] Squizzato S, Masiol M, Rich DQ, Hopke PK (2018). A long-term source apportionment of PM2.5 in New York state during 2005–2016. Atmos Environ.

[38] Zhao Y, Saleh R, Saliba G, Presto AA, Gordon TD, Drozd GT, Goldstein AH, Donahue NM, Robinson AL (2017). Reducing secondary organic aerosol formation from gasoline vehicle exhaust. Proc Natl Acad Sci.

[39] Hopke PK, Nadadur SS, Hollingsworth JW (2015). Reactive Ambient Particles. Air Pollution and Health Effects.

[40] Hopke PK (2008). New directions: reactive particles as a source of human health effects. Atmos Environ.

[41] Rich DQ, Zhang W, Lin S, Squizzato S, Thurston SW, van Wijngaarden E, Croft D, Masiol M, Hopke PK (2019). Triggering of cardiovascular hospital admissions by source specific fine particle concentrations in urban centers of New York state. Environ Int.

[42] Wang Y, Hopke PK, Utell MJ (2011). Urban-scale spatial-temporal variability of black carbon and winter residential wood combustion particles. Aerosol Air Qual Res.

[43] Croft DP, Zhang W, Lin S, Thurston SW, Hopke PK, Masiol M, Squizzato S, van Wijngaarden E, Utell MJ, Rich DQ (2019). The association between respiratory infection and air pollution in the setting of air quality policy and economic change. Ann Am Thorac Soc.

[44] D'Agostino RB, Vasan RS, Pencina MJ, Wolf PA, Cobain M, Massaro JM, Kannel WB (2008). General cardiovascular risk profile for use in primary care: the Framingham heart study. Circulation.

[45] Krall JR, Mulholland JA, Russell AG, Balachandran S, Winquist A, Tolbert PE, Waller LA, Sarnat SE (2017). Associations between source-specific fine particulate matter and emergency department visits for respiratory disease in four U.S. cities. Environ Health Perspect.

[46] Pirozzi CS, Jones BE, VanDerslice JA, Zhang Y, Paine R, Dean NC (2018). Short-term air pollution and incident pneumonia. A case-crossover study. Ann Am Thorac Soc.

[47] Horne BD, Joy EA, Hofmann MG, Gesteland PH, Cannon JB, Lefler JS, Blagev DP, Korgenski EK, Torosyan N, Hansen GI (2018). Short-term elevation of fine particulate matter air pollution and acute lower respiratory infection. Am J Respir Crit Care Med.

[48] Ciencewicki J, Jaspers I (2007). Air pollution and respiratory viral infection. Inhal Toxicol.

[49] Kwong JC, Schwartz KL, Campitelli MA, Chung H, Crowcroft NS, Karnauchow T, Katz K, Ko DT, McGeer AJ, McNally D (2018). Acute myocardial infarction after laboratory-confirmed influenza infection. N Engl J Med.

[50] Warren-Gash C, Hayward AC, Hemingway H, Denaxas S, Thomas SL, Timmis AD, Whitaker H, Smeeth L (2012). Influenza infection and risk of acute myocardial infarction in England and Wales: a CALIBER self-controlled case series study. J Infect Dis.

[51] Warren-Gash C, Smeeth L, Hayward AC (2009). Influenza as a trigger for acute myocardial infarction or death from cardiovascular disease: a systematic review. Lancet Infect Dis.

