# Supplementary material for: Changes in triggering of ST-elevation myocardial infarction by particulate air pollution in Monroe County, New York over time: a case-crossover study
Source: Environ Health. 2019 Sep 6;18:82. doi: 10.1186/s12940-019-0521-3 (PMC6728968; doi:10.1186/s12940-019-0521-3)
Supplement: Supplementary file 1 — Supplementary materials. (DOCX 393 kb) [file 12940_2019_521_MOESM1_ESM.docx]

**Additional file**

**Changes in Triggering of ST-elevation myocardial infarction by particulate air pollution in Monroe County, New York over time: a case-crossover study**

Meng Wang,^1^ Philip K. Hopke,^1,2^ Mauro Masiol,^1,2^ Sally W. Thurston,^3^ Scott Cameron,^5^

Frederick Ling,^5^ Edwin van Wijngaarden,^1,4^ Daniel Croft,^5^ Stefania Squizzato,^1,2^

Kelly Thevenet-Morrison,^1^ David Chalupa,^4^ David Q. Rich,^1,4,5^

| **Table S1.** Pearson correlation coefficients for hourly pollutant concentrations, temperature, and relative humidity (lag 0 of control periods), by time period | | | | | | | | | | | |  |
| --- | --- | --- | --- | --- | --- | --- | --- | --- | --- | --- | --- | --- |
| **Pollutant and Years** | **PM_2.5_** | **AMP** | **UFP** | **UFP**  **(11-50nm)** | **UFP**  **(50-100nm)** | **BC** | **SO_2_** | **O_3_** | **CO** | **Temperature** | **Relative humidity** | |
| **PM_2.5_** |  |  |  |  |  |  |  |  |  |  |  | |
| 2005-2016 | 1 | 0.66 | 0.21 | 0.13 | 0.34 | 0.59 | 0.20 | 0.05 | 0.36 | 0.19 | 0.09 | |
| 2005-2007 | 1 | 0.69 | 0.22 | 0.14 | 0.34 | 0.53 | 0.15 | 0.13 | 0.32 | 0.22 | 0.04 | |
| 2008-2013 | 1 | 0.66 | 0.19 | 0.1 | 0.34 | 0.64 | 0.13 | 0.07 | 0.30 | 0.25 | 0.06 | |
| 2014-2016 | 1 | 0.62 | 0.12 | 0.04 | 0.29 | 0.54 | 0.05 | 0.06 | 0.39 | 0.16 | 0.10 | |
| **AMP** |  |  |  |  |  |  |  |  |  |  |  | |
| 2005-2016 |  | 1 | 0.54 | 0.38 | 0.74 | 0.69 | 0.26 | -0.13 | 0.55 | 0.08 | 0.10 | |
| 2005-2007 |  | 1 | 0.53 | 0.37 | 0.73 | 0.71 | 0.15 | -0.16 | 0.56 | 0.14 | 0.08 | |
| 2008-2013 |  | 1 | 0.57 | 0.41 | 0.78 | 0.64 | 0.19 | -0.08 | 0.45 | 0.09 | 0.05 | |
| 2014-2016 |  | 1 | 0.29 | 0.13 | 0.60 | 0.66 | 0.05 | 0.03 | 0.41 | 0.24 | 0.06 | |
| **UFP** |  |  |  |  |  |  |  |  |  |  |  | |
| 2005-2016 |  |  | 1 | 0.96 | 0.78 | 0.46 | 0.39 | -0.24 | 0.50 | -0.13 | -0.03 | |
| 2005-2007 |  |  | 1 | 0.96 | 0.79 | 0.51 | 0.27 | -0.22 | 0.40 | -0.08 | -0.08 | |
| 2008-2013 |  |  | 1 | 0.96 | 0.79 | 0.39 | 0.32 | -0.18 | 0.47 | -0.21 | -0.08 | |
| 2014-2016 |  |  | 1 | 0.96 | 0.64 | 0.38 | 0.30 | -0.17 | 0.34 | 0 | -0.09 | |
| **UFP (11-50nm)** |  |  |  |  |  |  |  |  |  |  |  | |
| 2005-2016 |  |  |  | 1 | 0.59 | 0.35 | 0.38 | -0.21 | 0.42 | -0.15 | -0.07 | |
| 2005-2007 |  |  |  | 1 | 0.59 | 0.4 | 0.27 | -0.17 | 0.29 | -0.08 | -0.13 | |
| 2008-2013 |  |  |  | 1 | 0.59 | 0.28 | 0.32 | -0.15 | 0.41 | -0.24 | -0.12 | |
| 2014-2016 |  |  |  | 1 | 0.41 | 0.24 | 0.32 | -0.14 | 0.26 | -0.04 | -0.12 | |
| **UFP (50-100nm)** |  |  |  |  |  |  |  |  |  |  |  | |
| 2005-2016 |  |  |  |  | 1 | 0.58 | 0.3 | -0.25 | 0.55 | -0.06 | 0.07 | |
| 2005-2007 |  |  |  |  | 1 | 0.61 | 0.21 | -0.28 | 0.51 | -0.06 | 0.05 | |
| 2008-2013 |  |  |  |  | 1 | 0.5 | 0.22 | -0.19 | 0.47 | -0.09 | 0.03 | |
| 2014-2016 |  |  |  |  | 1 | 0.59 | 0.11 | -0.16 | 0.43 | 0.11 | 0.01 | |
| **BC** |  |  |  |  |  |  |  |  |  |  |  | |
| 2005-2016 |  |  |  |  |  | 1 | 0.16 | -0.37 | 0.62 | 0.09 | 0.23 | |
| 2005-2007 |  |  |  |  |  | 1 | 0.04 | -0.39 | 0.64 | 0.15 | 0.17 | |
| 2008-2013 |  |  |  |  |  | 1 | 0.05 | -0.34 | 0.49 | 0.1 | 0.21 | |
| 2014-2016 |  |  |  |  |  | 1 | -0.03 | -0.31 | 0.67 | 0.16 | 0.26 | |
| **SO_2_** |  |  |  |  |  |  |  |  |  |  |  | |
| 2005-2016 |  |  |  |  |  |  | 1 | -0.07 | 0.31 | -0.12 | -0.02 | |
| 2005-2007 |  |  |  |  |  |  | 1 | 0.04 | 0.01 | -0.03 | -0.14 | |
| 2008-2013 |  |  |  |  |  |  | 1 | 0 | 0.24 | -0.18 | -0.12 | |
| 2014-2016 |  |  |  |  |  |  | 1 | 0.01 | 0.01 | -0.13 | -0.05 | |
| **O_3_** |  |  |  |  |  |  |  |  |  |  |  | |
| 2005-2016 |  |  |  |  |  |  |  | 1 | -0.42 | 0.4 | -0.6 | |
| 2005-2007 |  |  |  |  |  |  |  | 1 | -0.39 | 0.39 | -0.52 | |
| 2008-2013 |  |  |  |  |  |  |  | 1 | -0.41 | 0.47 | -0.61 | |
| 2014-2016 |  |  |  |  |  |  |  | 1 | -0.35 | 0.29 | -0.63 | |
| **CO** |  |  |  |  |  |  |  |  |  |  |  | |
| 2005-2016 |  |  |  |  |  |  |  |  | 1 | -0.19 | 0.25 | |
| 2005-2007 |  |  |  |  |  |  |  |  | 1 | -0.07 | 0.19 | |
| 2008-2013 |  |  |  |  |  |  |  |  | 1 | -0.32 | 0.22 | |
| 2014-2016 |  |  |  |  |  |  |  |  | 1 | -0.16 | 0.25 | |
| **Temperature** |  |  |  |  |  |  |  |  |  |  |  | |
| 2005-2016 |  |  |  |  |  |  |  |  |  | 1 | -0.32 | |
| 2005-2007 |  |  |  |  |  |  |  |  |  | 1 | -0.27 | |
| 2008-2013 |  |  |  |  |  |  |  |  |  | 1 | -0.37 | |
| 2014-2016 |  |  |  |  |  |  |  |  |  | 1 | -0.28 | |

| **Table S2.** Rate of STEMI associated with each interquartile range increase in pollutant concentration (2005-2016) | | | | | | | | |
| --- | --- | --- | --- | --- | --- | --- | --- | --- |
| **Lag hours** | **Interquartile range** | | | | **No. of STEMI** | **Odds ratio** | **95%**  **confidence interval** | **p value** |
| **PM_2.5_**(µg/m^3^) |  | | | |  |  |  |  |
| 0 | 7.59 | | | | 858 | 1.03 | 0.94, 1.13 | 0.462 |
| 0-2 | 7.33 | | | | 848 | 1.05 | 0.95, 1.15 | 0.329 |
| 0-11 | 6.57 | | | | 855 | 1.02 | 0.93, 1.12 | 0.695 |
| 0-23 | 6.21 | | | | 854 | 1.01 | 0.91, 1.11 | 0.910 |
| 0-47 | 5.71 | | | | 848 | 0.98 | 0.89, 1.08 | 0.698 |
| 0-71 | 5.30 | | | | 833 | 0.96 | 0.86, 1.07 | 0.482 |
| **AMP**(particles/cm^3^) | |  | | |  |  |  |  |
| 0 | 698 | | | | 779 | 1.07 | 0.97, 1.18 | 0.163 |
| 0-2 | 688 | | | | 771 | 1.05 | 0.96, 1.16 | 0.298 |
| 0-11 | 693 | | | | 780 | 1.03 | 0.91, 1.15 | 0.666 |
| 0-23 | 670 | | | | 772 | 1.01 | 0.89, 1.14 | 0.913 |
| 0-47 | 602 | | | | 763 | 1.01 | 0.88, 1.15 | 0.917 |
| 0-71 | 543 | | | | 751 | 0.97 | 0.85, 1.11 | 0.645 |
| **UFP**(particles/cm^3^) | |  | | |  |  |  |  |
| 0 | 3702 | | | | 779 | 1.12 | 1.03, 1.22 | 0.008 |
| 0-2 | 3506 | | | | 771 | 1.11 | 1.02, 1.22 | 0.017 |
| 0-11 | 3265 | | | | 780 | 1.05 | 0.93, 1.18 | 0.407 |
| 0-23 | 2955 | | | | 772 | 1.00 | 0.88, 1.14 | 0.941 |
| 0-47 | 2568 | | | | 763 | 1.06 | 0.93, 1.22 | 0.382 |
| 0-71 | 2349 | | | | 751 | 1.00 | 0.86, 1.16 | 0.975 |
| **UFP 11-50nm**(particles/cm^3^) | | | |  |  |  |  |  |
| 0 | 2755 | | | | 779 | 1.13 | 1.04, 1.22 | 0.004 |
| 0-2 | 2551 | | | | 771 | 1.12 | 1.03, 1.21 | 0.011 |
| 0-11 | 2344 | | | | 780 | 1.03 | 0.92, 1.16 | 0.578 |
| 0-23 | 2151 | | | | 772 | 0.97 | 0.85, 1.12 | 0.716 |
| 0-47 | 1862 | | | | 763 | 1.04 | 0.90, 1.21 | 0.569 |
| 0-71 | 1682 | | | | 751 | 0.98 | 0.84, 1.15 | 0.819 |
| **UFP 50-100nm**(particles/cm^3^) | | | |  |  |  |  |  |
| 0 | 1161 | | | | 779 | 1.06 | 0.97, 1.15 | 0.192 |
| 0-2 | 1108 | | | | 771 | 1.06 | 0.97, 1.15 | 0.173 |
| 0-11 | 1118 | | | | 780 | 1.07 | 0.96, 1.20 | 0.240 |
| 0-23 | 1035 | | | | 772 | 1.05 | 0.94, 1.19 | 0.392 |
| 0-47 | 952 | | | | 763 | 1.09 | 0.95, 1.25 | 0.200 |
| 0-71 | 844 | | | | 751 | 1.02 | 0.89, 1.18 | 0.747 |
| **Black Carbon**(µg/m^3^) | | |  | |  |  |  |  |
| 0 | 0.44 | | | | 891 | 1.09 | 1.01, 1.18 | 0.034 |
| 0-2 | 0.43 | | | | 885 | 1.09 | 1.00, 1.18 | 0.046 |
| 0-11 | 0.42 | | | | 891 | 1.05 | 0.95, 1.16 | 0.337 |
| 0-23 | 0.39 | | | | 893 | 1.05 | 0.94, 1.16 | 0.405 |
| 0-47 | 0.35 | | | | 884 | 1.10 | 0.98, 1.23 | 0.122 |
| 0-71 | 0.33 | | | | 881 | 1.03 | 0.91, 1.17 | 0.630 |
| **SO_2_**(ppb) |  | | | |  |  |  |  |
| 0 | 1.77 | | | | 889 | 1.04 | 0.99, 1.09 | 0.150 |
| 0-2 | 2.20 | | | | 884 | 1.09 | 1.02, 1.18 | 0.019 |
| 0-11 | 2.27 | | | | 889 | 1.17 | 1.06, 1.29 | 0.002 |
| 0-23 | 2.30 | | | | 896 | 1.15 | 1.01, 1.30 | 0.029 |
| 0-47 | 2.19 | | | | 886 | 1.14 | 0.98, 1.33 | 0.090 |
| 0-71 | 2.22 | | | | 883 | 1.18 | 0.99, 1.41 | 0.071 |
| **O_3_** (ppb) |  | | | |  |  |  |  |
| 0 | 18.0 | | | | 884 | 0.89 | 0.77, 1.03 | 0.119 |
| 0-2 | 17.3 | | | | 872 | 0.90 | 0.78, 1.04 | 0.152 |
| 0-11 | 15.2 | | | | 888 | 0.92 | 0.80, 1.06 | 0.242 |
| 0-23 | 13.7 | | | | 893 | 0.92 | 0.79, 1.07 | 0.274 |
| 0-47 | 12.9 | | | | 887 | 0.87 | 0.73, 1.03 | 0.099 |
| 0-71 | 12.3 | | | | 883 | 0.90 | 0.75, 1.07 | 0.232 |
| **CO** (ppm) |  | | | |  |  |  |  |
| 0 | 0.23 | | | | 866 | 1.15 | 1.02, 1.29 | 0.020 |
| 0-2 | 0.23 | | | | 852 | 1.13 | 1.00, 1.28 | 0.047 |
| 0-11 | 0.21 | | | | 873 | 1.10 | 0.96, 1.27 | 0.171 |
| 0-23 | 0.22 | | | | 875 | 1.07 | 0.91, 1.25 | 0.418 |
| 0-47 | 0.21 | | | | 870 | 1.10 | 0.94, 1.30 | 0.235 |
| 0-71 | 0.21 | | | | 864 | 1.08 | 0.91, 1.29 | 0.367 |
| STEMI: ST-elevation myocardial infarction. Odds ratios were estimated from conditional logistic regression models adjusting for holiday and mean temperature and relative humidity during the same lag hour(s). | | | | | | | | |

| Table S3. Rate of STEMI associated with each interquartile range (3702 particles/cm^3^) increase in UFP concentration at lag hour 0 by patients’ characteristics | | | | |
| --- | --- | --- | --- | --- |
| Patients’ characteristics | Category | No. of STEMI | OR | 95%CI |
| Age | <65 years | 454 | 1.08 | (0.97 - 1.22) |
|  | ≥65 years | 321 | 1.18 | (1.04 - 1.34) |
|  |  |  |  |  |
| Sex | male | 554 | 1.16 | (1.05 - 1.29) |
|  | female | 225 | 1.04 | (0.89 - 1.21) |
|  |  |  |  |  |
| Smoking | Yes | 216 | 1.21 | (1.02 - 1.45) |
|  | No | 445 | 1.13 | (1.02 - 1.27) |
|  |  |  |  |  |
| Diabetes | Yes | 156 | 1.21 | (1.01 - 1.45) |
|  | No | 505 | 1.14 | (1.02 - 1.27) |
|  |  |  |  |  |
| Dyslipidemia | Yes | 354 | 1.20 | (1.05 - 1.37) |
|  | No | 305 | 1.12 | (0.98 - 1.27) |
|  |  |  |  |  |
| Heart Failure | Yes | 32 | 1.21 | (0.77 - 1.89) |
|  | No | 628 | 1.15 | (1.05 - 1.27) |
| STEMI: ST-elevation myocardial infarction; OR: odds ratio; 95%CI: 95% confidence interval. ORs were estimated from conditional logistic regression models adjusting for mean temperature and relative humidity during the same lag hour(s). | | | | |

| Table S4. Rate of STEMI associated with each interquartile range increase (3702 particles/cm^3^) in UFP concentration at lag hour 0 in the Before, During, and After Period, when separately including an interaction term between UFP and a patient characteristic. | | | | | |
| --- | --- | --- | --- | --- | --- |
| Age |  | <65 years | | >=65 years | |
|  |  | No. of STEMI | OR (95%CI) | No. of STEMI | OR (95%CI) |
|  | Before | 80 | 1.04 (0.89 - 1.21) | 84 | 1.14 (0.99 - 1.32) |
|  | During | 207 | 1.02 (0.85 - 1.22) | 119 | 1.13 (0.92 - 1.38) |
|  | After | 167 | 1.21 (1.02 - 1.44) | 118 | 1.34 (1.10 - 1.63) |
| Sex |  | Female | | Male | |
|  |  | No. of STEMI | OR (95%CI) | No. of STEMI | OR (95%CI) |
|  | Before | 60 | 1.03 (0.87 - 1.21) | 106 | 1.13 (0.98 - 1.31) |
|  | During | 95 | 0.98 (0.79 - 1.22) | 233 | 1.08 (0.90 - 1.29) |
|  | After | 70 | 1.16 (0.94 - 1.45) | 215 | 1.28 (1.08 - 1.51) |
| Smoking |  | No | | Yes | |
|  |  | No. of STEMI | OR (95%CI) | No. of STEMI | OR (95%CI) |
|  | Before | 92 | 1.11 (0.96 - 1.27) | 30 | 1.17 (0.95 - 1.45) |
|  | During | 168 | 1.06 (0.84 - 1.34) | 86 | 1.13 (0.88 - 1.46) |
|  | After | 185 | 1.23 (1.03 - 1.46) | 100 | 1.31 (1.06 - 1.62) |
| Diabetes |  | No | | Yes | |
|  |  | No. of STEMI | OR (95%CI) | No. of STEMI | OR (95%CI) |
|  | Before | 88 | 1.08 (0.93 - 1.26) | 34 | 1.18 (0.97 - 1.42) |
|  | During | 203 | 1.07 (0.86 - 1.34) | 51 | 1.16 (0.88 - 1.54) |
|  | After | 214 | 1.24 (1.05 - 1.46) | 71 | 1.34 (1.07 - 1.70) |
| Dyslipidemia |  | No | | Yes | |
|  |  | No. of STEMI | OR (95%CI) | No. of STEMI | OR (95%CI) |
|  | Before | 73 | 1.09 (0.93 - 1.27) | 49 | 1.16 (0.98 - 1.39) |
|  | During | 103 | 1.04 (0.81 - 1.33) | 151 | 1.11 (0.88 - 1.40) |
|  | After | 129 | 1.21 (1.00 - 1.46) | 154 | 1.30 (1.08 - 1.57) |
| Heart Failure |  | No | | Yes | |
|  |  | No. of STEMI | OR (95%CI) | No. of STEMI | OR (95%CI) |
|  | Before | 111 | 1.11 (0.97 - 1.28) | 11 | 1.19 (0.76 - 1.86) |
|  | During | 241 | 1.08 (0.87 - 1.34) | 13 | 1.15 (0.71 - 1.87) |
|  | After | 276 | 1.25 (1.07 - 1.47) | 8 | 1.33 (0.83 - 2.14) |

Figure S1. SO_2_ emissions from coal-fired power plants from 2005 to 2019 in western New York[1].

1. Squizzato S, Masiol M, Rich DQ, Hopke PK: **PM2.5 and gaseous pollutants in New York State during 2005–2016: Spatial variability, temporal trends, and economic influences**. *Atmospheric Environment* 2018, **183**:209-224.
